# Treatment of Crohn’s disease complicated with myelodysplastic syndrome via allogeneic hematopoietic stem cell transplantation: case report and literature review

**DOI:** 10.1007/s12328-014-0496-0

**Published:** 2014-05-22

**Authors:** Changmei Hu, Liang Lv, Deliang Liu, Jirong Huo

**Affiliations:** Department of Gastroenterology, Second Xiang Ya Hospital, Central South University, 139 Mid RenMin Road, Changsha, 410011 Hunan People’s Republic of China

**Keywords:** Crohn’s disease, Inflammatory bowel disease, Myelodysplastic syndrome, Hematopoietic stem cell transplantation, Allogeneic hematopoietic cell transplantation

## Abstract

Crohn’s disease (CD) is a chronic inflammatory disease of the gastrointestinal tract arising in individuals with genetic predisposing factors and abnormalities of the immune system. Myelodysplastic syndrome (MDS), an acquired clonal hematologic disorder, is characterized by peripheral blood cytopenia, dysplastic changes in several types of hematopoietic cells of the bone marrow and peripheral blood, and a high risk of transformation to acute leukemia. CD rarely occurs in combination with MDS, and MDS treatment with hematopoietic stem cell transplantation (HSCT) has not been frequently reported. We report the case of a 50-year-old Chinese male who presented with abdominal pain, diarrhea, and fatigue. CD was diagnosed by colonoscopy, imaging studies, and pathological examination. He was initially treated with mesalazine and prednisone and thereafter he presented with pancytopenia. MDS (RAEB-I) was diagnosed by bone marrow examination, and karyotyping revealed 47, XY, +8. The patient was treated with thalidomide, andriol, and decitabine. Allogeneic HSCT was performed with a human leukocyte antigen-matched sibling as the donor. The patient is currently well at 14 months after HSCT, without abdominal pain, diarrhea, or fatigue. HSCT may be a promising treatment option for patients with combined CD and MDS.

## Introduction

Crohn’s disease (CD) is a chronic inflammatory disease of the gastrointestinal (GI) tract arising in individuals with genetic predisposing factors and abnormalities of the immune system. It is characterized by periods of clinical relapse and remission, and its incidence in the general population is 1–10/10^5^. CD is a type of inflammatory bowel disease (IBD). Although its etiology and pathogenic mechanisms remain elusive, CD is thought to be caused by the interaction of multiple environmental, genetic, infectious, and immune factors. CD is frequently associated with extraintestinal manifestations. Previous studies have shown that the incidence of myelodysplastic syndrome (MDS) in patients with IBD ranges from 170−550/10^5^ [1, 2], which is higher than seen in the general population. It has been suggested that an association exists between CD and MDS. MDS, an acquired clonal hematologic disorder, is characterized by peripheral blood cytopenia, dysplastic changes in several types of hemopoietic cells in bone marrow and peripheral blood, and a high risk of transformation to acute leukemia. MDS has been associated with a variety of autoimmune phenomena, including systemic vasculitis syndrome, seronegative arthritis, relapsing polychondritis, etc. Some patients with MDS respond well to immunosuppressive agents, including corticosteroids, cyclosporine, and antithymocyte globulin [1]. CD rarely occurs in combination with MDS and, therefore, treatment with hematopoietic stem cell transplantation (HSCT) is not common for this condition. Here, we report the case of an adult patient with CD and MDS who was treated with allogeneic HSCT.

## Case presentation

**Fig. 1:**
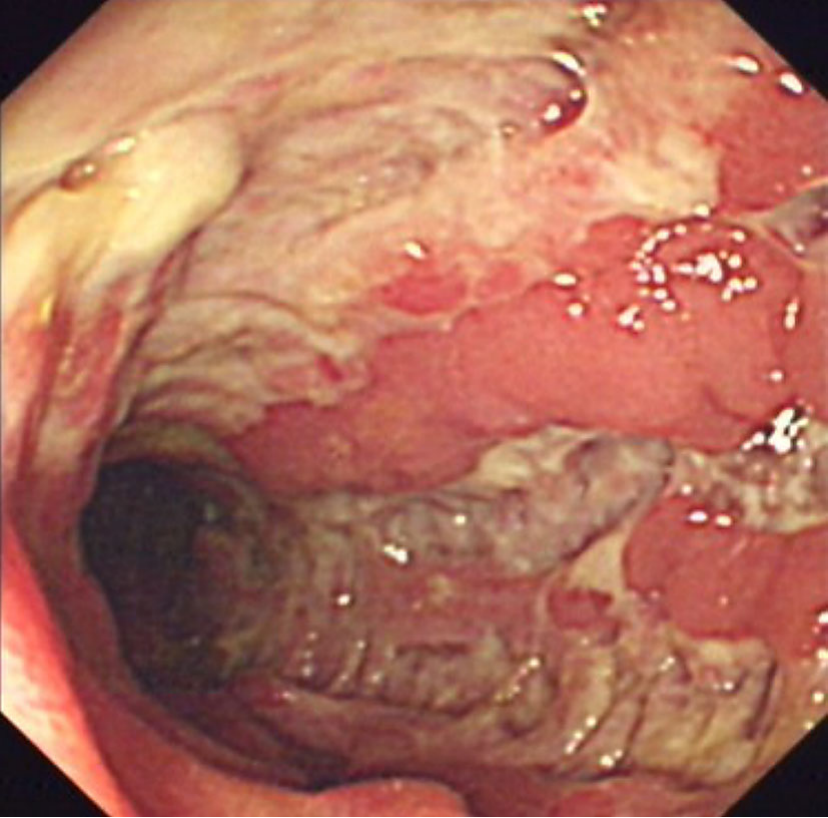
Colonoscopy. Deep and longitudinal multiple ulcers were observed at the colorectal mucosa. The mucosa plica between the ulcers were normal

**Fig. 2:**
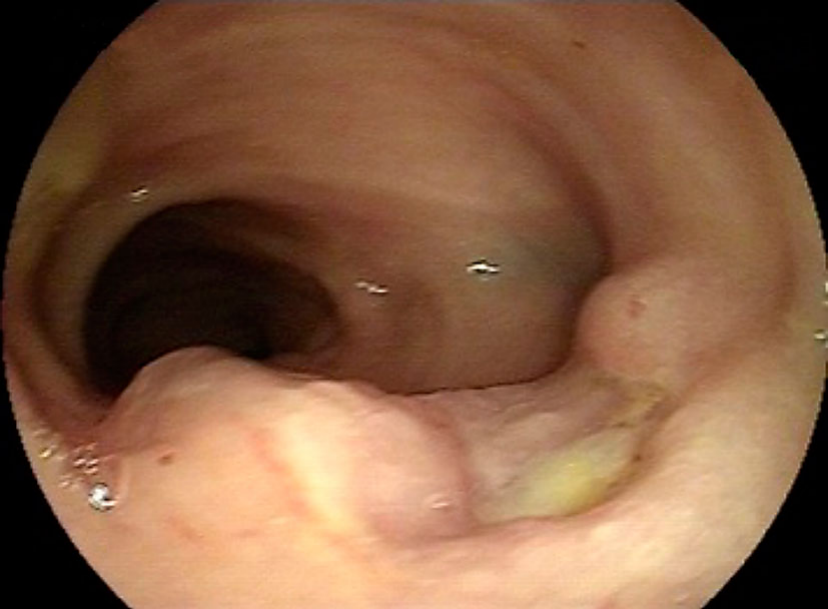
Double-balloon enteroscopy

**Fig. 3:**
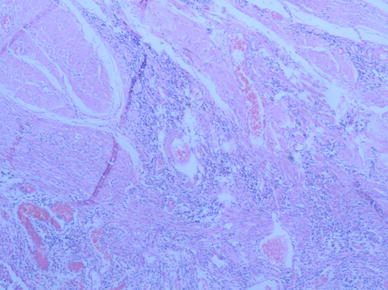
Pathological finding of colonic biopsy specimen. A slit-like ulcer reaching the muscularis propria was observed

**Fig. 4:**
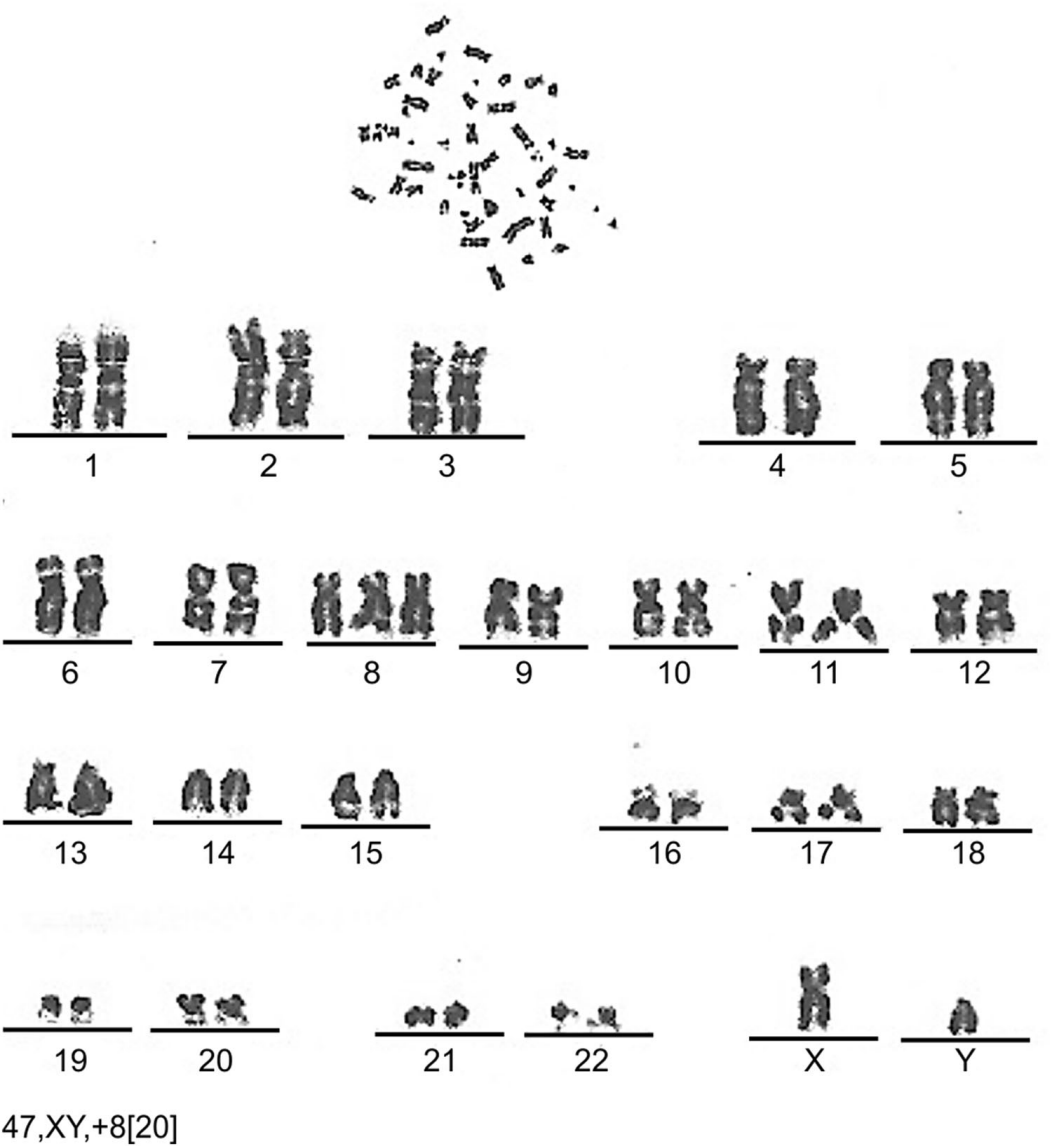
Karyotype analysis. A 47, XY, +8 (20) karyotype was observed

A 50-year-old Chinese male who had been experiencing persistent right lower abdominal pain, diarrhea, and fatigue for 6 months was admitted to our hospital. He occasionally had mild fever. The patient lost approximately 10 kg of weight over the 6-month period. He had a history of anal fistula for >1 year. Physical examination results were negative. A complete blood cell count analysis showed a white blood cell count of 12.3 × 10^9^/L, hemoglobin level of 109 g/L, and platelet count of 237 × 10^9^/L. The fecal occult blood test was positive. Serum albumin was 30 g/L, which was lower than the normal value. Barium enema examination confirmed the presence of mucosal disorders. Lesions showed segmental distribution. Colonoscopy showed mucosal sheet erosion and congestion in the ascending colon, with deep longitudinal ulcers, and double-balloon enteroscopy showed multiple longitudinal ulcers in the ileum (Figs. 1, 2). Imaging studies and pathological examination indicated a predominance of inflammatory cell invasion, and lymphoma was ruled out by immunohistochemical examination (Fig. 3) 1 month later. From the disease history, and results of the various tests, we confirmed a diagnosis of CD according to the diagnosis consensus of CD in Japan [3]. The patient was initially treated with mesalazine [1,000 mg, orally (PO), 4 times a day] and his symptoms improved. Four months later, prednisone was added [60 mg, (1 mg/kg PO), once a day] to the treatment regimen, after which his symptoms markedly improved. However, approximately 6 months later, the patient presented with pancytopenia (white blood cell count of 2.3 × 10^9^/L, hemoglobin level of 65 g/L, and platelet count of 55 × 10^9^/L), dizziness, and serious fatigue. Bone marrow examination confirmed a diagnosis of MDS (RAEB-I), and karyotyping revealed 47, XY, +8 (Fig. 4). Thalidomide (200 mg, PO, once a day) and andriol (40 mg, PO, twice a day) were administered in addition to mesalazine (1,000 mg, PO, once a day). The disease showed no obvious improvement. The patient continued to experience dizziness, fatigue, occasional abdominal pain, and diarrhea. After 9 months, the need for transfusion became evident, and the patient was started on induction chemotherapy with decitabine (20 mg/m^2^ once a day for 5 days, 2 cycles), after which a complete response was noted. Dizziness and fatigue significantly improved, but occasional abdominal pain and diarrhea persisted. Approximately 11 months after diagnosis, allogeneic HSCT was performed with a human leukocyte antigen (HLA)-matched sibling as the donor. The conditioning regimen was cyclophosphamide 60 mg/kg once a day for 2 days, total body irradiation 10 Gy and rabbit anti-human thymocyte globulin 2.5 mg/kg/days for 3 days. The bone marrow was reconstituted by the infusion of an unselected graft of 6.25 × 10^6^/kg CD34-positive stem cells. At 16 days after HSCT, the patient’s white blood cell count was >1.5 × 10^9^/L, and at 19 days, his platelet count was >20 × 10^9^/L. Methotrexate and cyclosporin A were used to prevent graft-versus-host disease. At 9 months after HSCT, the patient discontinued all immunosuppressive agents. At 14 months after HSCT, the patient was well with no symptoms of abdominal pain, diarrhea, or fatigue, and the anal fistula was cured. A chimerism study indicated 100 % donor chimerism at 25 months after diagnosis (Table 1).

**Table 1:** Chimerism study results

Genotype	Before transplantation	Donor	After transplantation
Sites			
D8S1179	12, 16	12, 13	12, 13
D21S11	29, 30	29, 29	29, 29
D7S820	11, 11	11, 11	11, 11
CSF1PO	11, 12	10, 12	10, 12
D3S1358	16, 17	14, 15	14, 15
TH01	6,10	9, 10	9, 10
D13S317	8, 8	8, 12	8, 12
D16S539	9, 9	12, 14	12, 14
D2S1338	19, 20	18, 23	18, 23
D19S433	14.2, 16.2	12,13.2	12, 13.2
VWA	14, 17	16, 18	16, 18
TPOX	8, 12	8, 8	8, 8
D18S51	14, 15	13, 19	13, 19
Amelogenin	X, Y	X, Y	X, Y
D5S818	7, 12	10, 11	10, 11
FGA	21, 23	22, 25	22, 25

Written informed consent was obtained from the patient for publication of this case report and any accompanying images. A copy of the written consent is available for review by the Editor-in-Chief of this journal.

## Discussion

A thorough review of the recent literature revealed that reports on CD combined with MDS treated with HSCT are extremely rare. One case of a 14-year-old male with CD since the age of 8 years has been reported. He was first administered prednisolone. After presenting with bloody diarrhea, oral prednisolone was discontinued and 6-mercaptopurine was commenced. Due to persistent diarrhea, weekly anti-tumor necrosis factor alpha (TNF-α) was started. The patient later became transfusion dependent and bone marrow studies showed MDS-erythrodysplasia with monosomy-7. Enzymatic studies confirmed TPMT heterozygosity. In 2004, HLA-matched sibling transplantation was performed. The patient is currently well 5 years and 8 months post-HSCT; his last chimerism study showed 100 % donor chimera [4]. Compared with our patient, immunosuppressive treatments were observed in this patient. In our case, the effects were not therapy related; however, both patients received the same treatment post-HSCT. To date, the mechanisms in both cases are unclear.

The pathogenic mechanisms of CD complicated by MDS are multiple, complex, and poorly understood. One hypothesis is concerned with the common immunological abnormalities. IBD is an autoimmune disease. Recent evidence suggests that MDS is associated with autoimmune diseases and carcinoid syndrome phenomena, such as relapsed polychondritis, Sjögren’s syndrome, rheumatoid arthritis, CD, and multiple vasculitis, may be observed in patients with MDS [2]. Patients with MDS also frequently present with abnormalities of the immune system and show a notable decrease in the T lymphocyte count. The Th1/Th2 ratio is also increased in these patients, whereas the counts and functions of CD8^+^ lymphocytes, natural killer cells, lymphokine-activated killer cells, and cytotoxic T lymphocytes are all lower than the established normal levels. In addition, lymphocytes secrete excessive amounts of interleukin 1, interleukin 6, and TNF-α. These cytokines play important roles in the pathogenesis of IBD. The production of interleukin and TNF-α induced by inflammatory reactions in patients with primary IBD may confer an increased risk for the development of MDS [1, 5, 6].

The second hypothesis to explain the pathogenic mechanism of CD complicated by MDS is based on common chromosomal abnormalities. Several gene loci have been used in the diagnosis of IBD, especially CD. Mutations in the *NOD2/CARD15* gene increase the risk of CD development. Alleles that confer susceptibility (e.g., IL23R) have been identified, and IBD is known to be associated with band 5q31 abnormalities on chromosome 5. Cytogenetic abnormalities in MDS have been extensively studied, and these may be significant for diagnosis, treatment, and prognostic evaluation. Although various multiple detectable chromosomal abnormalities have been described, the reported incidence of chromosomal abnormalities in patient with MDS combined with IBD is relatively low. Furthermore, patients harboring chromosomal abnormalities had a shorter interval between the developments of the 2 conditions than patients without chromosomal abnormalities [1, 2, 7]. Our patient showed a chromosomal constitution of 47, XY, +8, suggesting that chromosomal abnormalities may be an important pathogenic mechanism in the concomitant presentation of the 2 diseases. It is well known that trisomy 8 is a common chromosomal abnormality in MDS. It often manifests as GI lesions, including small bowel lesions [8].

Decreased phagocytosis of neutrophils in MDS patients is the third hypothesis proposed to explain the pathogenic mechanism of CD complicated by MDS. The use of iatrogenic immunosuppressant drugs in treatment of MDS and IBD increases patient vulnerability to intestinal and other infections and intestinal flora imbalance. Infection has strongly been associated with the pathogenesis and relapse of IBD and CD [9, 10].

HSCT is currently used for treatment of hematological malignancies, but has also been tested for non-malignant diseases and non-hematological diseases such as severe refractory autoimmune diseases and solid tumors. Normal hematopoietic stem cells are intravenously injected into the patient and settle in the bone marrow. The patient’s hematopoietic function is then restored through continuous self-renewal and differentiation of hematopoietic stem cells. This process may take 1−2 months, and the total recovery of immune function may take up to 6 months to 1 year [11]. HSCT can therefore both reconstitute the hematopoietic system and restore immune function.

Cases in which the pathogeneses of MDS and IBD are attributable to common immunological and chromosomal abnormalities can theoretically be cured through immune system reconstruction by HSCT, eliminating the need to clone abnormal chromosomes. The application of HSCT in the treatment of MDS is currently considered a sophisticated technology [12, 13]. Both allogeneic and autologous HSCT could effectively induce relief for patients in many severe refractory autoimmune diseases, including IBD [14].

**Fig. 5:**
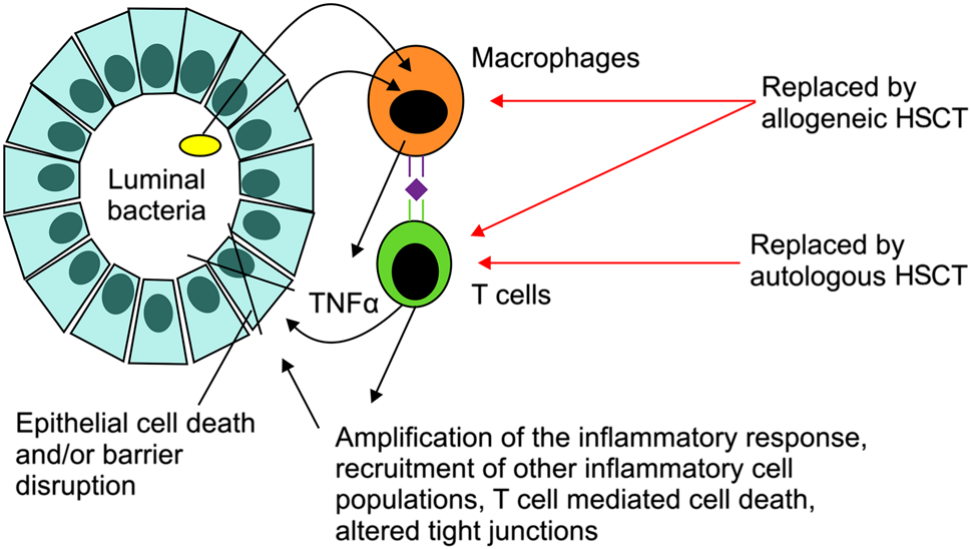
Pathogenesis of Crohn’s disease and proposed advantage of hematopoietic stem cell transplantation

The putative mechanism underlying the effect of treatment of IBD with HSCT is shown in Fig. 5.

Allogeneic HSCT may replace all recipient hemolymphatic cells with donor hemolymphatic cells harboring DNA sequences that do not predispose to CD, thereby alleviating CD. Autologous HSCT may replace hemolymphatic cells or their progenitor cells that generated the predisposing DNA sequence during ontogeny (e.g., T cell clones capable of recognizing the peptide) with healthy autologous hemolymphatic cells (e.g., T cell clones generated de novo in the thymus after HSCT that cannot recognize the peptide), thereby alleviating CD [14, 15]. The proposed mechanisms of autologous HSCT in the treatment of autoimmune diseases include immune suppression, immune resetting via repertoire replacement, and immune resetting via restoration of immune regulation [16]. Our patient with CD complicated by MDS showed satisfactory outcomes with allogeneic HSCT treatment.

## Conclusion

Several recent reports have described the favorable effects of HSCT in the treatment of patients with severe CD, including those with concomitant MDS [17–20]. Thus, HSCT may be a promising treatment option for patients with combined CD and MDS.

## References

[1] Harewood GC, Loftus EV, Tefferi A, Tremaine WJ, Sandborn WJ (1999). Concurrent inflammatory bowel disease and myelodysplastic syndrome. Inflamm Bowel Dis.

[2] Hebbar M, Kozlowski D, Wattel E, Mastrini S, Diévart M, Duclos B, Bonaz B, d’Almagne H, Belaiche J, Colombel JF, Fenaux P (1997). Association between myelodysplastic syndromes and inflammatory bowel diseases. Report of seven new cases and review of the literature. Leukemia.

[3] Ueno F, Matsui T, Matsumoto T, Matsuoka K, Watanabe M, Hibi T (2013). Guidelines Project Group of the Research Group of Intractable Inflammatory Bowel Disease subsidized by the Ministry of Health, Labour and Welfare of Japan and the Guidelines Committee of the Japanese Society of Gastroenterology. Evidence-based clinical practice guidelines for Crohn’s disease, integrated with formal consensus of experts in Japan. J Gastroenterol.

[4] Piccin A, Cortelazzo S, Rovigatti U, Bourke B, Smith OP (2010). Immunosuppressive treatments in Crohn’s disease induce myelodysplasia and leukaemia. Am J Hematol.

[5] Ditschkowski M, Einsele H, Schwerdtfeger R, Bunjes D, Trenschel R, Beelen DW, Elmaagacli AH (2003). Improvement of inflammatory bowel disease after allogeneic stem-cell transplantation. Transplantation.

[6] Lawrance IC (2012). Modifying T-cell trafficking to the intestinal as a potential management for inflammatory bowel disease. Expert Opin Investig Drugs.

[7] Cho JH (2008). Inflammatory bowel disease: genetic and epidemiologic considerations. World J Gastroenterol.

[8] Nakamura F, Watanabe T, Hori K, Ohara Y, Yamashita K, Tsuji Y, Ueda Y, Mikami S, Nakase H, Chiba T (2009). Simultaneous occurrence of inflammatory bowel disease and myelodysplastic syndrome due to chromosomal abnormalities in bone marrow cells. Digestion.

[9] Epling-Burnette PK, McDaniel J, Wei S, List AF (2012). Emerging immunosuppressive drugs in myelodysplastic syndromes. Expert Opin Emerg Drugs.

[10] Vanderploeg R, Panaccione R, Ghosh S, Rioux K (2010). Influences of intestinal bacteria in human inflammatory bowel disease. Infect Dis Clin North Am.

[11] Apperley J, Carreras E, Gluckman E, Gratwohl A, Masszi T. The 2008 revised edition of the EBMT-ESH handbook on haemopoietic stem cell transplantation. Institut de Recherche sur les leucemies et les Maladies du Sang Centre Hayem, Hopital Saint Louis-1, avenue Claude-Vellefaux, 75475 Paris Cedex 10, France. Ch. 2 Biological properties of haematopoietic stem cells. 35–44. Ch. 3 Immunogenetics of allogeneic HSCT. 47–91.

[12] Xu F, Deeg HJ (2012). Current status of allogeneic hematopoietic cell transplantation for MDS. Curr Pharm Des.

[13] Platzbecker U (2012). Allogeneic hematopoietic cell transplantation in patients with myelodysplastic syndromes. Semin Hematol.

[14] Leung Y, Geddes M, Storek J, Panaccione R, Beck PL (2006). Hematopoietic cell transplantation for Crohn’s disease; is it time?. World J Gastroenterol.

[15] Blondel-Kucharski F, Chircop C, Marquis P, Cortot A, Baron F, Gendre JP, Baron F, Colombel JF, Groupe d’Etudes Thérapeutique des Affections Inflammatoires Digestives (GETAID) (2001). Health-related quality of life in Crohn’s disease: a prospective longitudinal study in 231 patients. Am J Gastroenterol.

[16] Sullivan KM, Muraro P, Tyndall A (2010). Hematopoietic cell transplantation for autoimmune disease: updates from Europe and the United States. Biol Blood Marrow Transpl.

[17] Burt RK, Craig RM, Milanetti F, Quigley K, Gozdziak P, Bucha J, Testori A, Halverson A, Verda L, de Villiers WJ, Jovanovic B, Oyama Y (2010). Autologous nonmyeloablative hematopoietic stem cell transplantation in patients with severe anti-TNF refractory Crohn disease: long-term follow-up. Blood.

[18] Oyama Y, Craig RM, Traynor AE, Quigley K, Statkute L, Halverson A, Brush M, Verda L, Kowalska B, Krosnjar N, Kletzel M, Whitington PF, Burt RK (2005). Autologous hematopoietic stem cell transplantation in patients with refractory Crohn’s disease. Gastroenterology.

[19] Burt RK1, Verda L, Oyama Y, Statkute L, Slavin S. Non-myeloablative stem cell transplantation for autoimmune diseases. Springer Semin Immunopathol. 2004; 26:57–69. 10.1007/s00281-004-0162-6 15549303

[20] Hawkey CJ (2012). Stem cells as treatment in inflammatory bowel disease. Dig Dis.

